# Effects of six Chinese mind-body exercise therapies on glycolipid metabolism and vascular endothelial function in hypertensive patients

**DOI:** 10.1016/j.isci.2025.112682

**Published:** 2025-05-15

**Authors:** Haojie Li, Xinyu Lin, Xinglu Li, Nan Chen, Xie Wu

**Affiliations:** 1 School of Exercise and Health, Shanghai University of Sport, Shanghai, China; 2 Department of Rehabilitation, Chongming Hospital Affiliated to Shanghai University of Medicine and Health Sciences, Shanghai, China

**Keywords:** Cardiovascular medicine, Kinesiology

## Abstract

Hypertension adversely affects glycolipid metabolism and vascular function in aging populations. This network meta-analysis evaluated 36 randomized trials (*n* = 2875) comparing traditional mind-body exercises (Tai Chi, Baduanjin, Daoyinyangshengshu) for metabolic and vascular improvements. Analyses revealed Tai Chi most effectively raised high-density lipoprotein (HDL) (standardized mean differences [SMD] = 0.88), while Daoyinyangshengshu optimally reduced fasting glucose (SMD = −1.21) and endothelin-1 (SMD = −1.82) while increasing nitric oxide (SMD = 1.41). Baduanjin best lowered low-density lipoprotein (LDL) (SMD = −0.95), with other exercises improving cholesterol and triglycerides. The findings demonstrate that specific mind-body practices differentially enhance cardiometabolic health, with Tai Chi and Daoyinyangshengshu showing particular promise for comprehensive benefits. These evidence-based results support incorporating traditional exercises into hypertension management protocols to address both metabolic and vascular dysfunction.

## Highlights

• Reveals differential benefits of specific exercises on metabolic and vascular outcomes
• Provides evidence-based guidance for personalized exercise therapy selection
• Bridges traditional Chinese medicine with modern cardiovascular rehabilitation

## Graphical abstract

**Figure:**
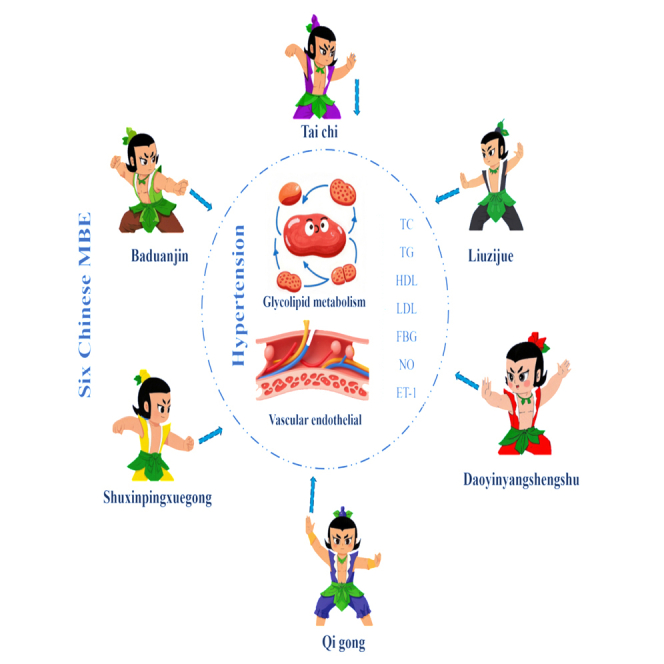

## Introduction

Hypertension in middle-aged and older adults has become a major global public health challenge, especially in China. According to the China Cardiovascular Health and Disease Report 2020,^1^ the prevalence of hypertension in middle-aged and older adults in China is as high as 55%, and the number of people with the disease is expected to increase to 400 million by 2030. Hypertension not only directly affects cardiovascular health but is also closely associated with many metabolic diseases, such as diabetes and obesity.^2^ Studies have shown that about 30% of hypertensive patients also have diabetes or metabolic syndrome, and these patients have twice the normal risk of heart disease.^3^ In addition, the long-term effects of hypertension include serious consequences such as stroke and kidney failure, which significantly reduce patients’ quality of life. Approximately 50% of people with hypertension will experience at least one cardiovascular event by the age of 60.^4^ Therefore, an in-depth study of the causes and interventions for hypertension in middle-aged and elderly people, especially combining traditional and modern medical approaches, is important for improving public health.

There is a complex and close relationship between hypertension and abnormalities of glucose and lipid metabolism, the latter of which is usually characterized by insulin resistance, elevated blood glucose levels, and dyslipidemia. Studies have shown that about 50% of middle-aged and elderly hypertensive patients also have diabetes or metabolic syndrome.^5^ Fasting blood glucose (FBG) levels in these patients often exceed 6.1 mmol/L, while low-density lipoprotein (LDL) levels are usually higher than 3.4 mmol/L, an abnormality that significantly increases the risk of cardiovascular disease. According to a study in the American Journal of Epidemiology, the risk of cardiovascular events increases by approximately 30% for every 1 mmol/L increase in LDL levels.^6^ In addition, disorders of glucolipid metabolism are also closely related to endothelial function impairment in hypertensive patients. Impairment of endothelial function is manifested by a decrease in nitric oxide (NO) synthesis and an increase in endothelin-1 (ET-1) levels, the latter leading to an enhanced vasoconstrictor response.^7^ One study found that systolic blood pressure (SBP) may increase by approximately 1 mmHg for every 1 pg/mL rise in ET-1, and in hypertensive patients, ET-1 levels may be twice as high as normal^8^ by 5%. This not only leads to a decrease in vasodilatory capacity but also affects hemodynamics, thereby exacerbating the development of hypertension.^9^ It has been established that improving glycolipid metabolism can help boost endothelial function. For example, in a 12-week clinical trial of a diet and exercise intervention for hypertensive patients, researchers found significant improvements in participants’ lipid markers, with reductions in cholesterol (total cholesterol, TC) and triglycerides (TGs) of 15% and 20%, respectively, as well as improvements in vascular endothelial function of up to 30%.^10^ Another study showed that hypertensive patients who performed regular aerobic exercise had a significant increase in NO levels and a significant decrease in ET-1 levels, resulting in improved endothelial function.^11^ Therefore, exploring the relationship between glycolipid metabolism and vascular endothelial function is important for the prevention and treatment of hypertension in middle-aged and elderly people.

Previous studies on hypertension have focused on pharmacologic interventions and aerobic exercise, but there are certain drawbacks. Although medication is effective in the short term, long-term dependence may lead to a variety of side effects, such as electrolyte imbalance, liver and kidney function impairment.^12^ Common aerobic exercises such as running and swimming are poorly adapted in the middle-aged and elderly population, especially those with pre-existing hypertension or other health problems.^13^ Middle-aged and older adults generally have decreased muscle mass and joint flexibility, and performing high-intensity exercise may significantly increase the risk of joint injuries, fractures, and cardiovascular events. Approximately 25% of middle-aged and older participants have reported sports injuries after such exercise.^14^ Moderate- to high-intensity exercise may cause sharp fluctuations in blood pressure, which is particularly dangerous for hypertensive individuals and may lead to an increased incidence of acute cardiovascular events such as myocardial infarction or stroke. In addition, the implementation of moderate- to high-intensity aerobic exercise often requires strong willpower and motivation, which may also be psychologically stressful and lead to decreased exercise compliance.

Traditional Chinese mind-body exercise therapies, such as Taichi, Baduanjin, Liuzijue, and Shuxinpingxuegong emphasize physical and mental harmony, slow movements, and breath regulation, which are particularly suitable for long-term adherence by middle-aged and elderly people. These therapies significantly improve physical and mental health by promoting qi and blood circulation, regulating the autonomic nervous system, and mental relaxation.^15^ Studies have shown that hypertensive patients who participate in Taichi training have an average reduction of 11 mmHg in systolic blood pressure and 6 mmHg in diastolic blood pressure, as well as improvements in heart rate and autonomic nervous system function.^16^ In addition, Baduanjin effectively enhances cardiovascular health by regulating autonomic nerves and reducing psychological stress. Studies have shown that after 8 weeks of Baduanjin training, participants had significant reductions in systolic and diastolic blood pressure, as well as significant improvements in mental health indicators.^15^^,^^17^ Compared with high-intensity aerobic exercise, traditional Chinese mind-body exercise therapy, with its lower intensity and slower tempo, puts less stress on the joints and cardiovascular system of hypertensive patients, thus reducing the risk of sports injuries and acute cardiovascular events, and plays an important role in overall health management, especially for hypertensive patients, and has significant clinical value.

In this study, we conducted the first systematic network meta-analysis of the effects of different Chinese traditional mind-body exercise therapies in improving blood metabolic molecules such as glucose-lipid metabolism and vascular endothelial secretory factor in middle-aged and elderly hypertensive patients by integrating 36 randomized controlled trials (RCTs), and comprehensively assessed the effect size of each therapy. In contrast to previous studies, many relevant studies have tended to focus on the effect of a single exercise therapy or on specific metabolic metrics and have lacked comprehensive comparisons and systematic assessments of different therapies. For example, some previous studies focused only on the effects of Taichi or Qigong on blood pressure or mental health. The results of the study provide a scientific basis for the effectiveness of traditional exercise therapy and suggest new ideas for the prevention and treatment of hypertension. This study hypothesizes that traditional Chinese mind-body exercise therapies can significantly improve glycolipid metabolism and vascular endothelial function in middle-aged and elderly hypertensive patients, and that different therapies may vary in their effects on these indicators. Through a network meta-analysis, this study systematically evaluates the comprehensive impact of different traditional Chinese mind-body exercise therapies on glycolipid metabolism and their improvement of vascular endothelial function in middle-aged and elderly hypertensive patients. It also compares the relative effects of each therapy on glycolipid metabolism and vascular endothelial function to identify which therapy may be more advantageous in specific indicators. The innovation of this study is to combine traditional Chinese medicine theories with modern medicine to provide a multidimensional perspective on the intervention of hypertension in middle-aged and elderly people, aiming to promote the development of relevant health policies and improve the overall health of middle-aged and elderly people. The findings of this study will provide novel insights and approaches for non-pharmacological interventions in middle-aged and elderly hypertensive patients, offer scientific evidence for the development and implementation of relevant health policies, and thereby enhance the overall health status of middle-aged and elderly individuals with hypertension.

## Results

### Literature search and selection

**Figure 1:**
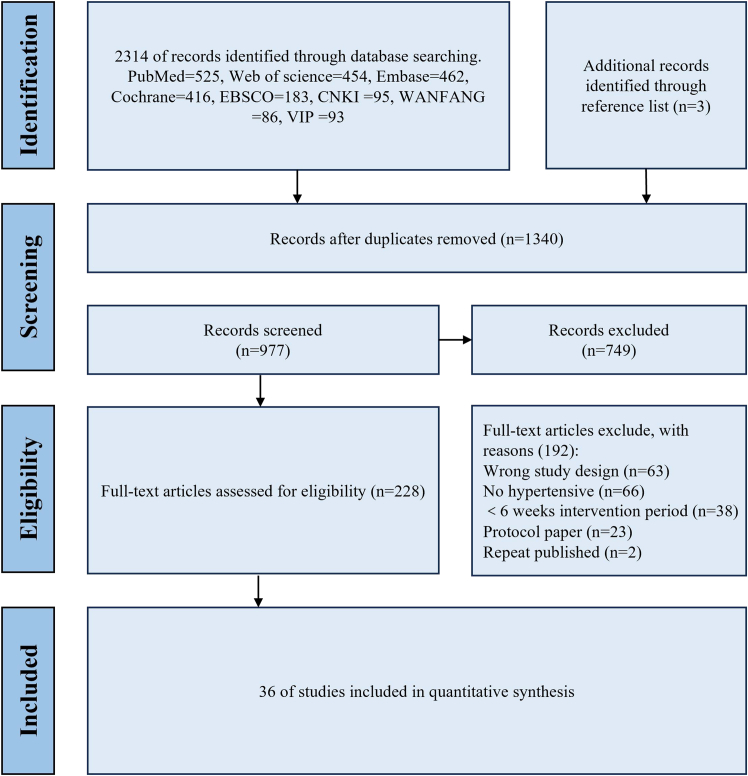
Literature search flowchart

A total of 2,317 potentially relevant documents were obtained through the electronic search, of which 2,314 were from databases and 3 originated from reference lists. After removing 1,340 duplicates, the remaining 977 documents entered the title and abstract screening stage, during which 749 documents were excluded. Based on the inclusion criteria, further full-text review was conducted to exclude 192 non-compliant documents, and 36 were finally included.^18^^,^^19^^,^^20^^,^^21^^,^^22^^,^^23^^,^^24^^,^^25^^,^^26^^,^^27^^,^^28^^,^^29^^,^^30^^,^^31^^,^^32^^,^^33^^,^^34^^,^^35^^,^^36^^,^^37^^,^^38^^,^^39^^,^^40^^,^^41^^,^^42^^,^^43^^,^^44^^,^^45^^,^^46^^,^^47^^,^^48^^,^^49^^,^^50^^,^^51^^,^^52^^,^^53^ The literature screening process is detailed in Figure 1.

### Literature characterization

This systematic review and network meta-analysis covered studies published from 2003 to 2023, and the experimental group included 1,456 hypertensive patients who received Tai Chi (696 patients), Baduanjin (469 patients), Liuzijue (37 patients), Shuxinpingxuegong (41 patients), Qigong (101 cases), and Daoyinyangshengshu (112 cases) interventions, and the control group contained 1,419 cases with an average participant age of 58.46 ± 7.86 years, totaling 2,875 experimental participants. In addition, several studies targeted patients with comorbidities such as type 2 diabetes or metabolic syndrome. Detailed characteristics of the included studies can be found in Table S1 (excel file containing additional data too large to fit in a PDF, related to STAR Methods). Six traditional Chinese mind-body exercise therapies (Tai Chi, Baduanjin, Liuzijue, Shuxinpingxuegong, Qigong, and Daoyinyangshengshu) were utilized in 36 randomized controlled trials and were compared with a no-exercise control group Comparisons were made. Tai Chi was the most frequently applied with 51.32%, followed by Baduanjin with 26.47%. The duration of each exercise ranged from 15 to 90 min, with a mean of 43.36 min. The average duration of the exercise intervention was 20.34 weeks, ranging from 8 to 48 weeks; more than 86.78% of the studies implemented a 12-week intervention. The average frequency of exercise per week was 3.8 sessions. Only three studies did not have a supervised intervention and used a combination of family interventions; the remaining studies were supervised interventions.

### Risk of bias assessment results and the grade of evidence

The median tool for the assessment of study quality and reporting in exercise (TESTEX) score of the included studies was 11 (see Table S2 for details). Among the studies reviewed, 5 studies (17%) reported physical activity levels in the control groups. Additionally, 21 studies (58%) conducted a valid intention-to-treat analysis, which is an essential methodological approach to minimize bias and ensure the robustness of the study results. Furthermore, 29 studies (69%) performed a blinded assessment of the primary outcome, thereby reducing the potential for subjective bias in the evaluation process. Lastly, 26 studies (72%) provided information on exercise attendance by the subjects, which is a critical factor in assessing the fidelity and effectiveness of the exercise interventions. The remaining TESTEX items were reported in more than 80% of the included studies, indicating a generally high level of adherence to the reporting standards outlined by the TESTEX scale. There are only indirect comparisons between mind-body exercise therapies, which results in a very low-quality rating for pairwise comparisons, the details of evidence evaluation utilizing GRADE is available.

### Direct pairwise meta-analyses

**Figure 2:**
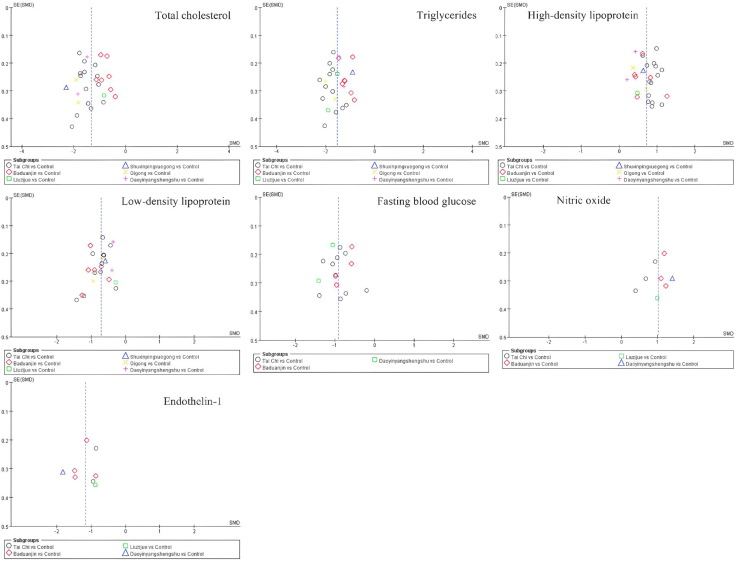
Funnel plot of total cholesterol (TC); triglycerides (TG); high-density lipoprotein (HDL); low-density lipoprotein (LDL); fasting blood glucose (FBG); nitric oxide (NO); endothelin-1 (ET-1) in pairwise meta-analysis

**Figure 3:**
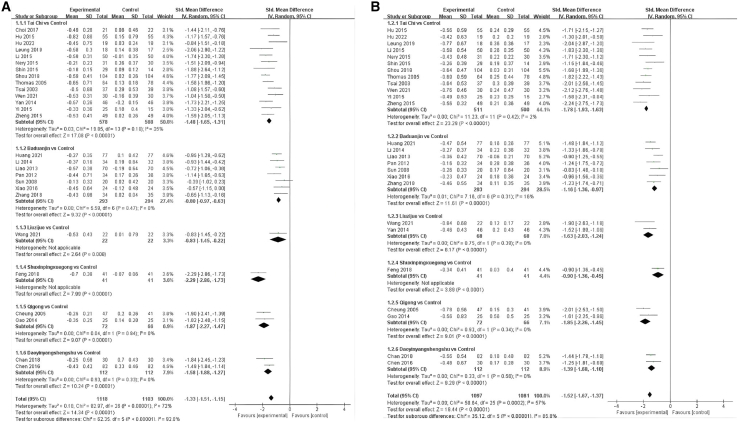
Forest plots for each pairwise comparison of total cholesterol (TC); triglycerides (TG) (A) is TC, (B) is TG, (−) indicates a negative number.

**Figure 4:**
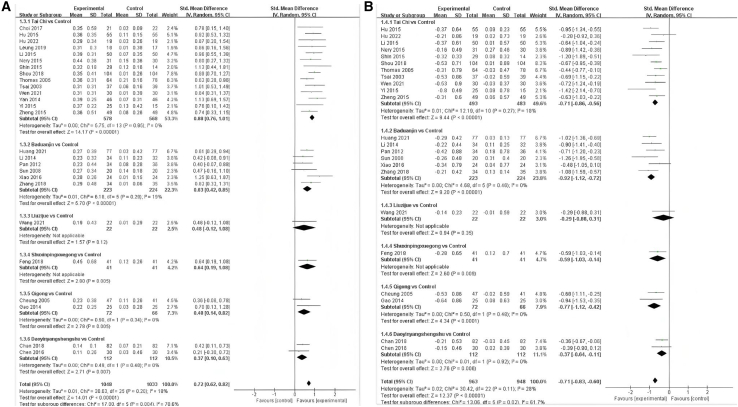
Forest plots for each pairwise comparison of high-density lipoprotein (HDL); low-density lipoprotein (LDL) (A) is HDL, (B) is LDL. (−) indicates a negative number.

**Figure 5:**
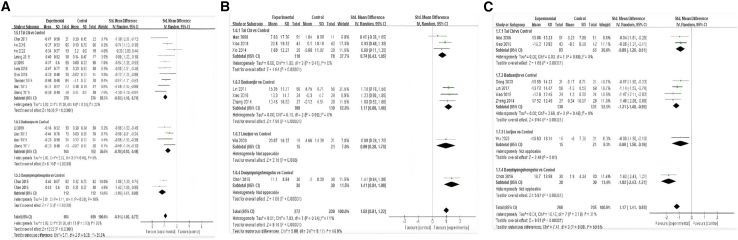
Forest plots for each pairwise comparison of fasting blood glucose (FBG); nitric oxide (NO); endothelin-1 (ET-1) (A) is FBG, (B) is NO, and (C) is ET-1. (−) indicates a negative number.

This net meta-analysis (NMA) began with a two-by-two meta-analysis, and the funnel plot is shown in Figure 2. The results are presented as a forest plot showing the effects of different exercise categories on TC, TG, HDL, LDL, FBG, NO, and ET-1(Figures 3, 4, and 5).

The results of the meta-analysis of TC, Tai Chi (SMD = −1.48, 95% CI: −1.65, −1.31, *p* < 0.00001, I^2^ = 35%, low evidence level), Baduanjin (SMD = −0.80, 95% CI: −0.97, −0.63, *p* < 0.00001, I^2^ = 0%, moderate evidence level), Liuzijue (SMD = −0.83, 95% CI: −1.45, −0.22, *p* = 0.008, low evidence level), Shuxinpingxuegong (SMD = −2.29, 95% CI: −2.86, −1.73, *p* < 0.00001, moderate evidence level), Qigong (SMD = −1.87, 95% CI: −2.27, −1.47 , *p* < 0.00001, I^2^ = 0%, low evidence level), and Daoyinyangshengshu (SMD = −1.58, 95% CI: −1.88, −1.27, *p* < 0.00001, I^2^ = 0%, low evidence level) all had a significant effect on reducing TC. The overall effect was SMD = −1.33, 95% CI: −1.51, −1.15, *p* < 0.00001, I^2^ = 72% (Figure 3A).

The results of the meta-analysis of TG, Tai Chi (SMD = −1.78, 95% CI: −1.93, −1.63], *p* < 0.00001, I^2^ = 2%, moderate evidence level), Baduanjin (SMD = −1.16, 95% CI: −1.36, −0.97, *p* < 0.00001, I^2^ = 16%, low evidence level), Liuzijue (SMD = −1.63, 95% CI: −2.03, −1.24, *p* < 0.00001, I^2^ = 0%, moderate evidence level), Shuxinpingxuegong (SMD = −0.90, 95% CI: 1.36, −0.45, *p* = 0.0001, low evidence level), Qigong (SMD = −1.85, 95% CI: 2.26, −1.45, *p* < 0.00001, I^2^ = 0%, moderate evidence level), and Daoyinyangshengshu (SMD = −1.39, 95% CI: −1.68, −1.10, *p* < 0.00001, I^2^ = 0%, low evidence level) all significantly improved TG. The overall effect was SMD = −1.52, 95% CI: −1.67, −1.37, *p* < 0.00001, I^2^ = 57% (Figure 3B).

The results of the meta-analysis of HDL, Tai Chi (SMD = 0.88, 95% CI: 0.76, 1.01, *p* < 0.00001, I^2^ = 0%), low evidence level, Baduanjin (SMD = 0.63, 95% CI: 0.42, 0.85, *p* < 0.00001, I^2^ = 19%, moderate evidence level), Liuzijue (SMD = 0.48, 95% CI: 0.12, 1.08, *p* = 0.12, low evidence level), Shuxinpingxuegong (SMD = 0.64, 95% CI: 0.19, 1.08, *p* = 0.005, low evidence level), Qigong (SMD = 0.48, 95% CI: 0.14, 0.82, *p* = 0.005, I^2^ = 0%, moderate evidence level), and Daoyinyangshengshu (SMD = 0.37, 95% CI: 0.10, 0.63, *p* = 0.007 I^2^ = 0%, very low evidence level) all significantly elevated HDL levels, with an overall effect of SMD = 0.72, 95% CI: 0.62, 0.82, *p* < 0.00001, I^2^ = 18% (Figure 4A).

The results of meta-analysis of LDL, Tai Chi (SMD = −0.71, 95% CI: −0.86, −0.56, *p* < 0.00001, I^2^ = 18%, low evidence level), Baduanjin (SMD = −0.92, 95% CI: −1.12, −0.72, *p* < 0.00001, I^2^ = 0%, moderate eidence level), Liuzijue (SMD = −0.29, 95% CI: −0.88, 0.31, *p* = 0.35, moderate evidence level), Shuxinpingxuegong (SMD = −0.59, 95% CI: −1.03, −0.14, *p* = 0.009, low evidence level), Qigong (SMD = −0.77, 95% CI: −1.12, −0.42, *p* < 0.00001, I^2^ = 0%, low evidence level), and Daoyinyangshengshu (SMD = −0.37, 95% CI: −0.64, −0.11, *p* = 0.006, I^2^ = 0%, very low evidence level) all significantly reduced LDL levels, with an overall effect of SMD = −0.71, 95% CI: −0.83, −0.60, *p* < 0.00001, I^2^ = 28% (Figure 4B).

The meta-analysis of FBG resulted in an overall effect for Tai Chi (SMD = −0.92, 95% CI: −1.10, −0.74, *p* < 0.00001, I^2^ = 22%, moderate evidence level), Baduanjin (SMD = −0.70, 95% CI: −0.93, −0.48], *p* < 0.00001, I^2^ = 0%, low evidence level), and Daoyinyangshengshu (SMD = −1.16, 95% CI: −1.47, −0.85, *p* < 0.00001, I^2^ = 10%, low evidence level) all significantly reduced FBG levels with an overall effect of SMD = −0.91, 95% CI: −1.05, −0.77, *p* < 0.00001, I^2^ = 30% (Figure 5A).

NO meta-analysis results, Tai Chi (SMD = 0.74, 95% CI: 0.43, 1.05, *p* < 0.00001, I^2^ = 0%, low evidence level), Baduanjin (SMD = 1.17, 95% CI: 0.88, 1.46, *p* < 0.00001, I^2^ = 0%, moderate evidence level), Liuzijue (SMD = 0.99, 95% CI: 0.28, 1.70, *p* = 0.006, low evidence level), and Daoyinyangshengshu (SMD = 1.41, 95% CI: 0.84, 1.98, *p* < 0.00001, moderate evidence level) all significantly increased NO levels, with an overall effect of SMD = 1.02, 95% CI: 0.81, 1.22, *p* < 0.00001, I^2^ = 11% (Figure 5B).

For ET-1 analysis results, Tai Chi (SMD = −0.88, 95% CI: −1.26, −0.51, *p* < 0.00001, I^2^ = 0%, low evidence level), Baduanjin (SMD = −1.21, 95% CI: 1.48, −0.95, *p* < 0.00001, I^2^ = 0%, low evidence level), Liuzijue (SMD = −0.88, 95% CI: 1.58, −0.18, *p* = 0.01, low evidence level), and Daoyinyangshengshu (SMD = −1.82, 95% CI: −2.43, −1.21, *p* < 0.00001, moderate evidence level) all significantly reduced ET-1 levels, with an overall effect of SMD = −1.17, 95% CI: −1.41, −0.93, *p* < 0.00001, I^2^ = 31% (Figure 5C).

### Network meta-analysis

**Figure 6:**
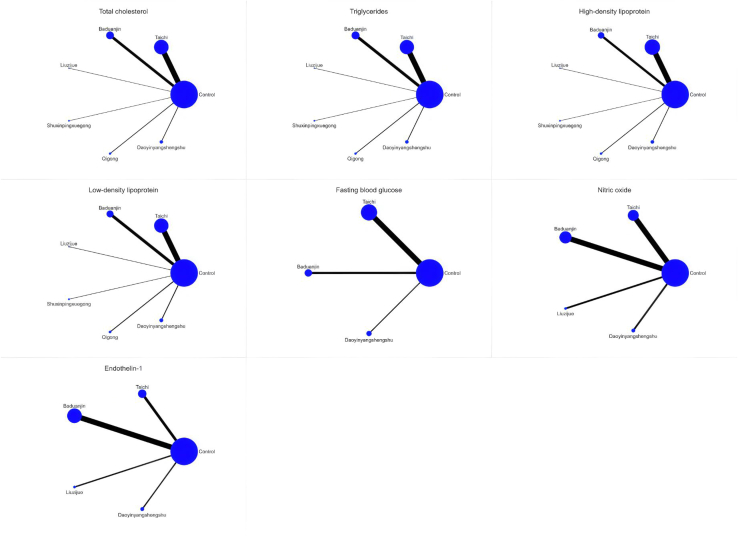
Illustrates the NMA plots for TC, TG, HDL, LDL, FBG, NO, and ET-1, which assessed the effects of six traditional Chinese mind-body exercise therapies

**Figure 7:**
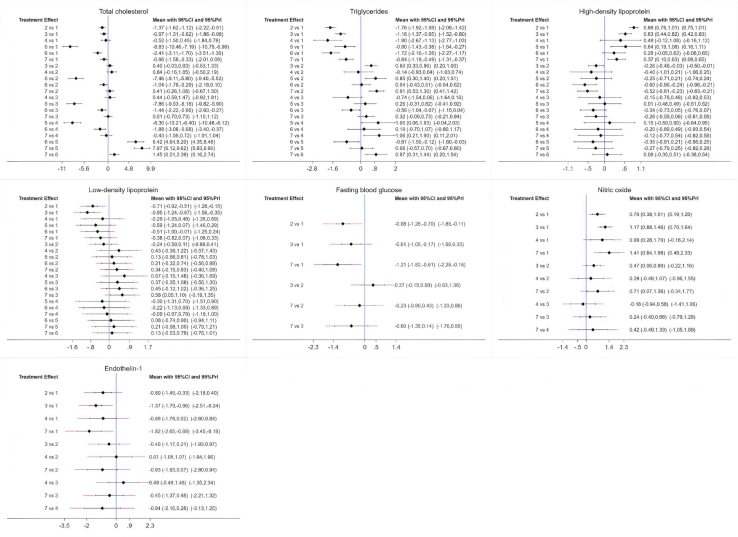
Forest plots of eligible comparisons of total cholesterol (TC); triglycerides (TG); high-density lipoprotein (HDL); low-density lipoprotein (LDL); fasting blood glucose (FBG); nitric oxide (NO); endothelin-1 (ET-1) (−) indicates a negative number.

**Figure 8:**
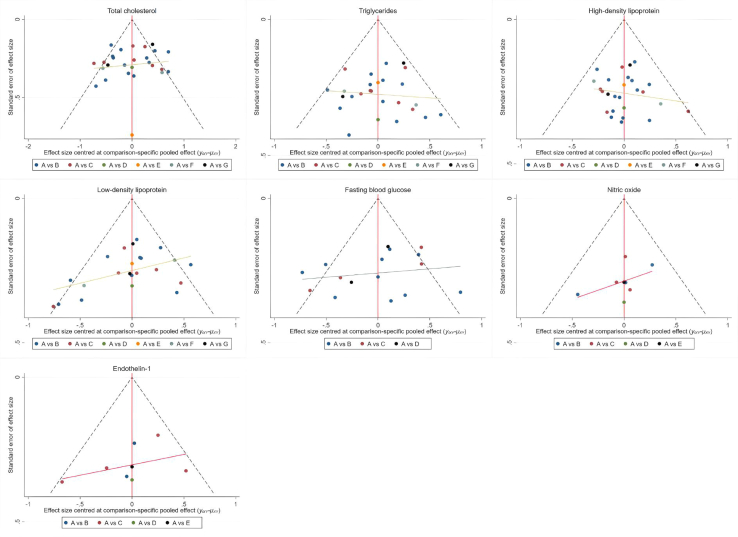
The funnel plot graphics of total cholesterol (TC); triglycerides (TG); high-density lipoprotein (HDL); low-density lipoprotein (LDL); fasting blood glucose (FBG); nitric oxide (NO); endothelin-1 (ET-1) in NMA

**Figure 9:**
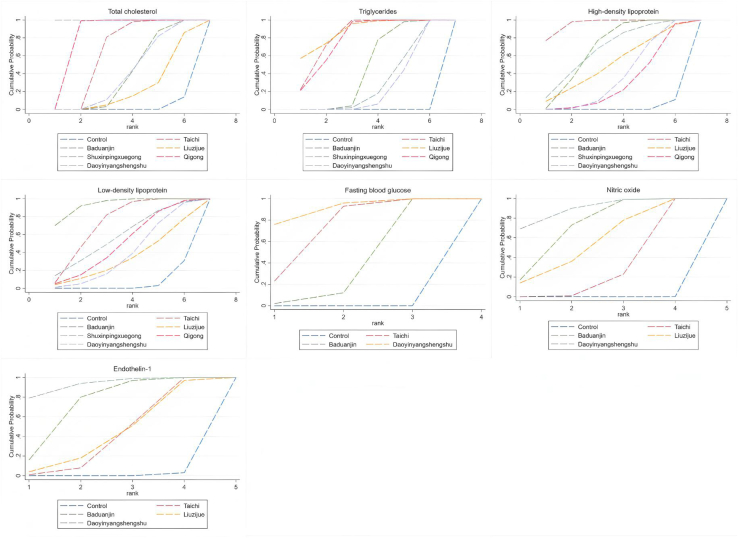
Area under the curve for cumulative ranking probability of each intervention on total cholesterol (TC); triglycerides (TG); high-density lipoprotein (HDL); low-density lipoprotein (LDL); fasting blood glucose (FBG); nitric oxide (NO); endothelin-1 (ET-1)

Figure 6 illustrates the NMA plots for TC, TG, HDL, LDL, FBG, NO, and ET-1, which assessed the effects of six traditional Chinese mind-body exercise therapies. The size of the nodes in the graphs reflects the sample size of each exercise therapy, and the thickness of the lines between the nodes indicates the number of studies comparing these interventions. Tai Chi is the most widely studied intervention, whereas Daoyinyangshengshu, Qigong, and Shuxinpingxuegong have been less frequently studied. Network contribution plots for direct and indirect comparisons and the number of studies for each direct comparison are detailed. Forest plots of the combined results for TC, TG, HDL, LDL, FBG, NO, and ET-1, including 95% CI and 95% PrI, are shown in Figure 7. Funnel plots to test for publication bias and small-sample effects in the NMA are shown in Figure 8, and the graphs are largely symmetrical, indicating that publication bias or small sample effects were less likely and passed the consistency assessment. The SUCRA probabilities for each intervention are shown in Figure 9, with larger SUCRA values indicating that the intervention ranked higher in the network, reflecting a higher probability of being the best intervention.

#### Aggregate estimates of TC

Pooled TC estimates are shown in Table 1. Tai Chi (SMD = −1.37, 95% CI: −1.62, −1.12, *p* = 0, low evidence level), Baduanjin (SMD = −0.97, 95% CI: −1.31, −0.62, *p* = 0, moderate evidence level), Shuxinpingxuegong (SMD = −8.83, 95% CI: −10.46, −7.19, *p* = 0, moderate evidence level), Qigong (SMD = −2.41, 95% CI: −3.11, −1.70, *p* = 0, low evidence level). Daoyinyangshengshu (SMD = −0.96, 95% CI: −1.58, −0.33, *p* = 0, low evidence level) demonstrated Improvements in TC compared with control. The effectiveness of different Chinese traditional mind-body exercise therapies in improving TC in hypertensive patients were ranked as Shuxinpingxuegong (SUCRA = 100), Qigong (SUCRA = 83.2), Taichi (SUCRA = 63.3), Daoyinyangshengshu (SMD = −0.96, 95% CI: −1.58, −0.33, *p* = 0), Daoyinyangshengshu (SUCRA = 39.4), Baduanjin (SUCRA = 39), Liuzijue (SUCRA = 22.7), and all interventions outperformed the control group (SUCRA = 2.4) Table 2.

**Table 1:** Network meta-analysis matrix of primary and secondary outcomes

**Total cholesterol**						

Taichi	0.40 (−0.03,0.83)	0.84 (−0.16,1.85)	−7.46 (−9.11,−5.80)	−1.04 (−1.79,−0.29)	0.41 (−0.26,1.08)	1.37 (1.12,1.62)
−0.40 (−0.83,0.03)	Baduanjin	0.44 (−0.59,1.47)	−7.86 (−9.53,−6.18)	−1.44 (−2.22,−0.66)	0.01 (−0.70,0.73)	0.97 (0.62,1.31)
−0.84 (−1.85,0.16)	−0.44 (−1.47,0.59)	Liuzijue	−8.30 (−10.21,−6.40)	−1.88 (−3.08,−0.68)	−0.43 (−1.59,0.72)	0.52 (−0.45,1.50)
7.46 (5.80,9.11)	7.86 (6.18,9.53)	8.30 (6.40,10.21)	Shuxinpingxuegong	6.42 (4.64,8.20)	7.87 (6.12,9.62)	8.83 (7.19,10.46)
1.04 (0.29,1.79)	1.44 (0.66,2.22)	1.88 (0.68,3.08)	−6.42 (−8.20,−4.64)	Qigong	1.45 (0.51,2.39)	2.41 (1.70,3.11)
−0.41 (−1.08,0.26)	−0.01 (−0.73,0.70)	0.43 (−0.72,1.59)	−7.87 (−9.62,−6.12)	−1.45 (−2.39,−0.51)	Daoyinyangshengshu	0.96 (0.33,1.58)
**−1.37 (−1.62,−1.12)**	**−0.97 (−1.31,−0.62)**	−0.52 (−1.50,0.45)	**−8.83 (−10.46,−7.19)**	**−2.41 (−3.11,−1.70)**	**−0.96 (−1.58,−0.33)**	Control

**Triglycerides**						

Taichi	0.60 (0.33,0.86)	−0.14 (−0.93,0.64)	0.85 (0.30,1.40)	0.04 (−0.43,0.51)	0.91 (0.53,1.30)	1.76 (1.59,1.92)
−0.60 (−0.86,−0.33)	Baduanjin	−0.74 (−1.54,0.06)	0.26 (−0.31,0.82)	−0.56 (−1.04,−0.07)	0.32 (−0.09,0.73)	1.16 (0.95,1.37)
0.14 (−0.64,0.93)	0.74 (−0.06,1.54)	Liuzijue	1.00 (0.06,1.93)	0.19 (−0.70,1.07)	1.06 (0.21,1.90)	1.90 (1.13,2.67)
−0.85 (−1.40,−0.30)	−0.26 (−0.82,0.31)	−1.00 (−1.93,−0.06)	Shuxinpingxuegong	−0.81 (−1.50,−0.12)	0.06 (−0.57,0.70)	0.90 (0.38,1.43)
−0.04 (−0.51,0.43)	0.56 (0.07,1.04)	−0.19 (−1.07,0.70)	0.81 (0.12,1.50)	Qigong	0.87 (0.31,1.44)	1.72 (1.28,2.16)
−0.91 (−1.30,−0.53)	−0.32 (−0.73,0.09)	−1.06 (−1.90,−0.21)	−0.06 (−0.70,0.57)	−0.87 (−1.44,−0.31)	Daoyinyangshengshu	0.84 (0.49,1.19)
**−1.76 (−1.92,−1.59)**	**−1.16 (−1.37,−0.95)**	**−1.90 (−2.67,−1.13)**	**−0.90 (−1.43,−0.38)**	**−1.72 (−2.16,−1.28)**	**−0.84 (−1.19,−0.49)**	Control

**High-density lipoprotein**						

Taichi	−0.26 (−0.48,−0.03)	−0.40 (−1.01,0.21)	−0.25 (−0.71,0.21)	−0.60 (−0.96,−0.24)	−0.52 (−0.81,−0.23)	−0.88 (−1.01,−0.76)
0.26 (0.03,0.48)	Baduanjin	−0.15 (−0.78,0.48)	0.01 (−0.48,0.49)	−0.34 (−0.73,0.05)	−0.26 (−0.59,0.06)	−0.63 (−0.82,−0.44)
0.40 (−0.21,1.01)	0.15 (−0.48,0.78)	Liuzijue	0.15 (−0.59,0.90)	−0.20 (−0.89,0.49)	−0.12 (−0.77,0.54)	−0.48 (−1.08,0.12)
0.25 (−0.21,0.71)	−0.01 (−0.49,0.48)	−0.15 (−0.90,0.59)	Shuxinpingxuegong	−0.35 (−0.91,0.21)	−0.27 (−0.79,0.25)	−0.64 (−1.08,−0.19)
0.60 (0.24,0.96)	0.34 (−0.05,0.73)	0.20 (−0.49,0.89)	0.35 (−0.21,0.91)	Qigong	0.08 (−0.35,0.51)	−0.28 (−0.62,0.05)
0.52 (0.23,0.81)	0.26 (−0.06,0.59)	0.12 (−0.54,0.77)	0.27 (−0.25,0.79)	−0.08 (−0.51,0.35)	Daoyinyangshengshu	−0.37 (−0.63,−0.10)
**0.88 (0.76,1.01)**	**0.63 (0.44,0.82)**	0.48 (−0.12,1.08)	**0.64 (0.19,1.08)**	0.28 (−0.05,0.62)	**0.37 (0.10,0.63)**	Control

**Low-density lipoprotein**						

Taichi	−0.24 (−0.59,0.11)	0.43 (−0.36,1.22)	0.13 (−0.56,0.81)	0.21 (−0.32,0.74)	0.34 (−0.15,0.83)	0.71 (0.51,0.92)
0.24 (−0.11,0.59)	Baduanjin	0.67 (−0.15,1.48)	0.37 (−0.35,1.08)	0.45 (−0.12,1.02)	0.58 (0.05,1.10)	0.95 (0.67,1.24)
−0.43 (−1.22,0.36)	−0.67 (−1.48,0.15)	Liuzijue	−0.30 (−1.31,0.70)	−0.22 (−1.13,0.69)	−0.09 (−0.97,0.79)	0.29 (−0.48,1.05)
−0.13 (−0.81,0.56)	−0.37 (−1.08,0.35)	0.30 (−0.70,1.31)	Shuxinpingxuegong	0.08 (−0.74,0.90)	0.21 (−0.58,1.00)	0.59 (−0.07,1.24)
−0.21 (−0.74,0.32)	−0.45 (−1.02,0.12)	0.22 (−0.69,1.13)	−0.08 (−0.90,0.74)	Qigong	0.13 (−0.53,0.79)	0.51 (0.01,1.00)
−0.34 (−0.83,0.15)	−0.58 (−1.10,−0.05)	0.09 (−0.79,0.97)	−0.21 (−1.00,0.58)	−0.13 (−0.79,0.53)	Daoyinyangshengshu	0.38 (−0.07,0.82)
**−0.71 (−0.92,−0.51)**	**−0.95 (−1.24,−0.67)**	−0.29 (−1.05,0.48)	−0.59 (−1.24,0.07)	**−0.51 (−1.00,−0.01)**	−0.38 (−0.82,0.07)	Control

**Fasting blood glucose**						

Taichi		0.37 (−0.15,0.89)		−0.23 (−0.90,0.43)	0.98 (0.70,1.26)	
−0.37 (−0.89,0.15)		Baduanjin		−0.60 (−1.35,0.14)	0.61 (0.17,1.05)	
0.23 (−0.43,0.90)		0.60 (−0.14,1.35)		Daoyinyangshengshu	1.21 (0.61,1.82)	
**−0.98 (−1.26,−0.70)**		**−0.61 (−1.05,−0.17)**		**−1.21 (−1.82,−0.61)**	Control	

**Nitric oxide**						

Taichi		0.47 (0.05,0.90)	0.29 (−0.48,1.07)	0.71 (0.07,1.36)	−0.70 (−1.01,−0.38)	
−0.47 (−0.90,−0.05)		Baduanjin	−0.18 (−0.94,0.58)	0.24 (−0.40,0.88)	−1.17 (−1.46,−0.88)	
−0.29 (−1.07,0.48)		0.18 (−0.58,0.94)	Liuzijue	0.42 (−0.49,1.33)	−0.99 (−1.70,−0.28)	
−0.71 (−1.36,−0.07)		−0.24 (−0.88,0.40)	−0.42 (−1.33,0.49)	Daoyinyangshengshu	−1.41 (−1.98,−0.84)	
**0.70 (0.38,1.01)**		**1.17 (0.88,1.46)**	**0.99 (0.28,1.70)**	**1.41 (0.84,1.98)**	Control	

**Endothelin-1**						

Taichi		−0.48 (−1.17,0.21)	0.01 (−1.05,1.07)	−0.93 (−1.93,0.07)	0.89 (0.33,1.45)	
0.48 (−0.21,1.17)		Baduanjin	0.49 (−0.49,1.48)	−0.45 (−1.37,0.48)	1.37 (0.96,1.79)	
−0.01 (−1.07,1.05)		−0.49 (−1.48,0.49)	Liuzijue	−0.94 (−2.16,0.28)	0.88 (−0.02,1.78)	
0.93 (−0.07,1.93)		0.45 (−0.48,1.37)	0.94 (−0.28,2.16)	Daoyinyangshengshu	1.82 (0.99,2.65)	
**−0.89 (−1.45,−0.33)**		**−1.37 (−1.79,−0.96)**	−0.88 (−1.78,0.02)	**−1.82 (−2.65,−0.99)**	Control	

Effects are expressed as effect sizes (95% Cl) between interventions. Bolding indicates a significant effect of the exercise intervention. (−) indicates a negative number.

**Table 2:** Ranking of exercise interventions in order of effectiveness

Total cholesterol		Triglycerides		High-density lipoprotein	
Treatment	SUCRA	Treatment	SUCRA	Treatment	SUCRA
Shuxinpingxuegong	100	Liuzijue	87.4	Taichi	95.8
Qigong	83.2	Taichi	82.2	Baduanjin	67.9
Taichi	63.3	Qigong	78.9	Shuxinpingxuegong	67.3
Daoyinyangshengshu	39.4	Baduanjin	46.7	Liuzijue	50.9
Baduanjin	39	Shuxinpingxuegong	29.9	Daoyinyangshengshu	36.8
Liuzijue	22.7	Daoyinyangshengshu	24.9	Qigong	29.5
Control	2.4	Control	0	Control	1.8

Fasting blood glucose		Nitric oxide		Endothelin-1	
Treatment	Treatment	Treatment	SUCRA	Treatment	SUCRA
Daoyinyangshengshu	90.4	Daoyinyangshengshu	89.4	Daoyinyangshengshu	93
Taichi	71.8	Baduanjin	72.2	Baduanjin	73.2
Baduanjin	37.8	Liuzijue	57	Liuzijue	42.6
Control	0.1	Taichi	31.2	Taichi	40.6
		Control	0.1	Control	0.7

Low-density lipoprotein	
Treatment	SUCRA
Baduanjin	93.4
Taichi	71.6
Shuxinpingxuegong	57.8
Qigong	49.9
Daoyinyangshengshu	38.2
Liuzijue	33.3
Control	5.7

#### Aggregate estimates of TG

Tai Chi (SMD = −1.76, 95% CI: −1.92, −1.59, *p* = 0, moderate evidence level), Baduanjin (SMD = −1.16, 95% CI: −1.37, −0.95, *p* = 0, low evidence level), Liuzijue (SMD = −1.90, 95% CI: −2.67, −1.13, *p* = 0, moderate evidence level), Shuxinpingxuegong (SMD = −0.90, 95% CI: −1.43, −0.38, *p* = 0, low evidence level), Qigong (SMD = −1.72, 95% CI: −2.16, −1.28, *p* = 0, moderate evidence level), Daoyinyangshengshu (SMD = −0.84, 95% CI: −1.19, −0.49, *p* = 0, low evidence level) demonstrated improvements in TG compared with control (Table 1). The order of effectiveness of different Chinese traditional mind-body exercise therapies to improve TG in hypertensive patients was Liuzijue (SUCRA = 87.4), Taichi (SUCRA = 82.2), Qigong (SUCRA = 78.9), Baduanjin (SUCRA = 46.7), Shuxinpingxuegong (SUCRA = 29.9), Daoyinyangshengshu (SUCRA = 24.9), and all interventions were superior to the control group (SUCRA = 0) (Table 2).

#### Summary estimation for HDL

(Table 1) In terms of HDL improvement, Tai Chi (SMD = 0.88, 95% CI: 0.76, 1.01, *p* = 0, low evidence level), Baduanjin (SMD = 0.63, 95% CI: 0.44, 0.82, *p* = 0, moderate evidence level), Shuxinpingxuegong (SMD = −0.64, 95% CI: 0.19, 1.08, *p* = 0, low evidence level) demonstrated improvements. Daoyinyangshengshu (SMD = 0.37, 95% CI: 0.10, 0.63, *p* < 0.05, very low evidence level) demonstrated improvements in HDL compared with control. different Chinese traditional mind-body exercise therapies to improve hypertension. The effectiveness of different Chinese traditional mind-body exercise therapies in improving HDL in patients was ranked as Taichi (SUCRA = 95.8), Baduanjin (SUCRA = 67.9), Shuxinpingxuegong (SUCRA = 67.3), Liuzijue (SUCRA = 50.9), Daoyinyangshengshu (SUCRA = 36.8), and Qiuyinyangshengshu (SUCRA = 36.8), and the effectiveness of different Chinese traditional mind-body exercise therapies in improving HDL in patients with hypertension. 36.8), Qigong (SUCRA = 29.5), and all interventions were superior to the control group (SUCRA = 1.8) (Table 2).

#### Summary estimation of LDL

Tai Chi (SMD = −0.71, 95% CI: −0.92, −0.51, *p* = 0, low evidence level), Baduanjin (SMD = −0.95, 95% CI: −1.24, −0.67, *p* = 0, moderate evidence level), Qigong (SMD = −0.51, 95% CI: −1.00, −0.01, *p* < 0.05, low evidence level) demonstrated improvements in LDL compared with control. demonstrated improvements in LDL compared with control (Table 1). The effectiveness of different Chinese traditional mind-body exercise therapies to improve LDL in hypertensive patients was ranked as Baduanjin (SUCRA = 93.4), Taichi (SUCRA = 71.6), Shuxinpingxuegong (SUCRA = 57.8), Qigong (SUCRA = 49.9), Daoyinyangshengshu (SUCRA = 38.2), and Liuzijue (SUCRA = 33.3), and all interventions were better than the control group (SUCRA = 5.7) (Table 2).

#### Aggregate estimation of FBGs

(Table 1) Tai Chi (SMD = −0.98, 95% CI: −1.26, −0.70, *p* = 0, moderate vidence Level, Baduanjin (SMD = −0.61, 95% CI: −1.05, −0.17, *p* < 0.05, low evidence level), Daoyinyangshengshu (SMD = −1.21, 95% CI: −1.82, −0.61, *p* = 0.05, low evidence level) demonstrated improvements in FBG compared with control.The effectiveness of different Chinese traditional mind-body exercise therapies to improve FBG in hypertensive patients was ranked Daoyinyangshengshu (SUCRA = 90.4), Taichi (SUCRA = 71.8), and Baduanjin (SUCRA = 37.8), and all the interventions were superior to the control group (SUCRA = 0.1) (Table 2).

#### Summary estimates of NO

Tai Chi (SMD = 0.70, 95% CI: 0.38, 1.01, *p* = 0, low evidence level), Baduanjin (SMD = 1.17, 95% CI: 0.88, 1.46, *p* = 0, moderate evidence level), Liuzijue (SMD = 0.99, 95% CI: 0.28, 1.70, *p* < 0.05, low evidence level), Daoyinyangshengshu (SMD = 1.41, 95% CI: 0.84, 1.98, *p* = 0.05, moderate evidence level) demonstrated improvements in NO compared with control. Daoyinyangshengshu (SMD = 1.41, 95% CI: 0.84, 1.98, *p* = 0.05, low evidence level) demonstrated improvements in NO compared with control (Table 1). The effectiveness of different Chinese traditional mind-body exercise therapies in improving NO in hypertensive patients was ranked as follows Daoyinyangshengshu (SUCRA = 89.4), Baduanjin (SUCRA = 72.2), Liuzijue (SUCRA = 57), and Taichi (SUCRA = 31.2), and all interventions were superior to control (SUCRA = 0.1) (Table 2).

#### Summary estimates for ET-1

(Table 1) Tai Chi (SMD = −0.89, 95% CI: −1.45, −0.33, *p* < 0.05, low evidence level), Baduanjin (SMD = −1.37, 95% CI: −1.79, −0.96, *p* = 0, low evidence level), Daoyinyangshengshu (SMD = −1.82, 95% CI: −2.65, −0.99, *p* = 0, moderate evidence level) demonstrated improvements in ET-1 compared with control. The effectiveness of different Chinese traditional mind-body exercise therapies in improving ET-1 in hypertensive patients was ranked as Daoyinyangshengshu (SUCRA = 93), Baduanjin (SUCRA = 73.2), Liuzijue (SUCRA = 42.6), Taichi (SUCRA = 40.6), Control (SUCRA = 0.7) (Table 2).

### Minimally contextualized framework

Use control as the reference group. The intervention was classified as “category 0”, with no clinical difference compared to the intervention group, “category 1”, which was clinically superior to the intervention group and “category 2”, which is superior to one or more other intervention groups. Category 0: indicates no clinical difference compared to the intervention group. Then, a secondary classification is performed based on the differences between the interventions. Using the interventions with the smallest effect size in the first category as a reference, the more effective interventions were divided into the second category. The interventions were then grouped into high and low reliability categories according to hierarchical classification and the consistency of the classification was tested by ranking results. In this study, ensuring that the highest ranked interventions were one of the most effective ones (see Table S3) (excel file containing additional data too large to fit in a PDF, related to STAR Methods).

## Discussion

The Cochrane risk of bias assessment tool was used in this study, and the results showed that 31 studies were at low risk of bias, 2 were at moderate risk, and 3 were at high risk of bias. This indicates that the overall risk of bias in this study was low and the results possessed a high level of confidence. In addition, the quality of evidence for the outcome indicators was assessed using the GRADE framework, and the quality of evidence for most of the indicators was low to moderate. To ensure the robustness of the meta-analysis results, we performed a funnel plot analysis and found that there may be a certain amount of publication bias, which may lead to underestimation or overestimation of certain intervention effects. To control for this effect, we performed subgroup analyses and found that traditional mind-body exercise therapy had significant effects in improving vascular health and metabolic markers regardless of risk of bias, type of intervention, or duration of intervention. Given the significant clinical efficacy of Baduanjin and Daoyinyangshengshu in improving TC and HDL, these therapies should be prioritized in future clinical practice. Future research should further investigate the combined effects of these therapies with other lifestyle interventions, such as diet and psychological support, to optimize the comprehensive management of hypertensive patients.

### Effects on glycolipid metabolism

Our study found that traditional Chinese mind-body exercise therapy significantly improved glycolipid metabolic indices in hypertensive patients. Specifically, Taichi reduced the effect size of TC (SMD = −1.37, 95% CI: −1.62, −1.12) and had a significant improvement in TG (SMD = −1.76, 95% CI: −1.92, −1.59), and significantly increased HDL levels (SMD = 0.88, 95% CI: 0.76, 1.01). These findings are consistent with the study by Li et al. which found that Taichi reduced TG by 1.5 mmol/L while increasing HDL levels by approximately 0.8 mmol/L.^54^ Baduanjin achieved an improvement in TG (SMD = −1.16, 95% CI: −1.37, −0.95).^55^ Shuxinpingxuegong showed more significant improvement in TC (SMD = −8.83, 95% CI: −10.46, −7.19) than other exercise therapies, which may be related to its specific respiratory regulation and physical and mental relaxation mechanisms, which in turn promote lipid metabolism more effectively.^56^

Compared with previous studies, the results of our study differed in some metrics from data from other exercise therapies. For example, Lin et al. found that Taichi did not improve FBG as well as Baduanjin (SMD = −0.6 vs. −1.1), whereas in our study it was shown that Taichi outperformed Baduanjin in reducing FBG (SMD = −0.98 vs. −0.61). This difference may stem from differences in sample characteristics, intervention duration, and exercise intensity. From a mechanistic point of view, conventional exercise therapy contributes to the reduction of FBG and TG levels by promoting insulin sensitivity, increasing muscle uptake of glucose, and improving fatty acid oxidation efficiency.^57^ The mechanism of this improvement is associated with the activation of the insulin signaling pathway and the AMPK (adenylate-activated protein kinase) pathway, which promotes the homeostasis of systemic energy metabolism.^58^ In addition, these therapies indirectly improve glucolipid metabolism by modulating the autonomic nervous system and reducing the secretion of stress hormones (e.g., cortisol).^59^ Future research should focus on the long-term effects of these therapies, particularly their impact on the incidence of cardiovascular events and quality of life. Additionally, exploring the differential effects of various therapies on different individuals (e.g., varying ages, genders, and disease severities) is essential for achieving more precise clinical decision-making.

### Effects on vascular endothelial function

The results of this study showed that patients who participated in traditional Chinese mind-body exercise therapy demonstrated significant improvements in endothelial function indices (e.g., NO and ET-1). Daoyinyangshengshu performed well in this study, with the magnitude of the NO enhancement and ET-1 reduction effect at the forefront of the therapies. Specifically, Daoyinyangshengshu significantly improved NO levels in hypertensive patients (SMD = 1.41, 95% CI: 0.84, 1.98) and lowered ET-1 (SMD = −1.82, 95% CI: −2.65, −0.99). The increase in NO improves vasodilatation, which lowers blood pressure, while the decrease in ET-1 reduces blood pressure. decrease in ET-1 reduced the vasoconstrictor response. These results are consistent with the study by Liu et al.,^60^ who found that patients who participated in Taichi training for a long period of time had elevated NO levels of up to 30% and a reduction in ET-1 of up to 20%. This supports the findings of our study that traditional Chinese mind-body exercise therapy plays a significant role in the regulation of vascular endothelial function.

However, part of the literature shows that other therapies such as Taichi have a weaker effect on NO improvement (SMD = 0.7),^41^ which is inconsistent with the significantly elevated effect of Taichi found in this study (SMD = 0.70, 95% CI: 0.38, 1.01). This difference may be related to the frequency of training, the duration of the intervention, and the initial blood pressure status of the patients. The study also suggests that breathing regulation and relaxation training helps to increase endothelial nitric oxide synthase (eNOS) expression in vascular endothelial cells, which in turn increases NO synthesis, while training in breathing regulation and mental relaxation helps to decrease ET-1 secretion, which alleviates vasoconstrictor responses, and this dual mechanism of action helps to improve vascular health and stabilize blood pressure, and also enhances vascular elasticity and diastolic function.^61^

### Limitations of the study

A limitation of this study was the screening of randomized controlled trials (RCTs) by including in the analysis all studies that explicitly referred to “randomized group” or “controlled design” in the text, without further limiting their methodological details (e.g., random sequence generation mode, allocation concealment, etc.). This relaxed criterion was intended to broaden the coverage of the literature.

## Resource availability

### Lead contact

Further information and requests for resources should be directed to and will be fulfilled by the lead contact, Xie Wu (wuxie_sus@163.com).

### Materials availability

This study did not generate new unique reagents.

### Data and code availability

• The dataset generated for this paper is available from https://doi.org/10.6084/m9.figshare.28463360.v2.
• The software and code used in this study are available in https://handbook-5-1.cochrane.org/, https://www.stata.com/ and https://endnote.com/downloads.
• Autonomous generated codes used in the methods section of the text are available at https://doi.org/10.6084/m9.figshare.28909031.v1. Any additional information required to reanalyze the data reported in this paper is available from the lead contact upon request.

## Acknowledgments

This work was supported by Key Medical Discipline Project in Chongming District, Shanghai, China.

## Author contributions

All authors contributed to the conception and design of the study. Data collection and analysis were performed by H.L. The first draft of the manuscript was written by X.W., and all authors reviewed and approved the final manuscript.

## Declaration of interests

The authors declare no competing interests.

## STAR★Methods

### Key resources table

**Table:** 

REAGENT or RESOURCE	SOURCE	IDENTIFIER
**Deposited data**		

Literature summary statistics	Figshare: https://doi.org/10.6084/m9.figshare.28463360.v2	Li
Self-generated method code	Research Methodology Generation	https://doi.org/10.6084/m9.figshare.28909031.v1

**Software and algorithms**		

Cochrane 5.1	Higgins et al.	https://handbook-5-1.cochrane.org/
Stata17.0	StataCorp LLC	https://www.stata.com/
Endnote X9.1	Thomson Scientific	https://endnote.com/downloads

### Experimental model and study participant details

#### Experimental model

As this study is a systematic review and meta-analysis, it utilizes existing literature rather than employing the experimental models prevalent in the life sciences.

#### Ethics approval and consent to participate

Ethical approval was not sought for this study because the study does not contain any animal or direct human participants for experiments.

### Method details

#### Registration

The study protocol for this NMA was registered with the PROSPERO International Registry of Systematic Reviews (registration number: **CRD42024593391**) and was strictly in accordance with the Preferred Reporting Items for Systematic Review and Meta -Analysis, PRISMA) statement for reporting (Hutton et al.).

#### Literature search strategy

This study searched the following databases: PubMed, Web of Science, Cochrane, EBSCO, Embase, China Knowledge Network (CNKI), Wanfang Digital Journals (WANFANG), and Chinese Science and Technology Journal Database (VIP). In PubMed/Cochrane and Embase, the searches used relevant terms from the MeSH and Emtree vocabularies. For the Chinese databases CNKI, WANFANG and VIP, we combined subject term and free word searches. The specific search strategy for each database is detailed in Table S4. The search strategy was designed based on the PICOS framework:(P) Study population: hypertensive patients; (I) Interventions: Taichi, Baduanjin, Liuzijue, Shuxinpingxuegong, Qigong, Daoyinyangshengshu; (C) Control group: no exercise or light stretching; (O) outcome metrics: TC, TG, HDL, LDL, FBG, NO, and ET-1; (S) study type: RCTs. in addition, further eligible studies were sought by hand-searching the reference lists of eligible literature and relevant reviews.

#### Eligibility criteria

This study strictly screened studies that met the following criteria:(1) age ≥45 years (Di Francescomarino et al.); (2) the study population was hypertensive (SBP/DBP >130/80 mmHg) (Whelton et al.); (3) the use of Taichi, Baduanjin, Liuzijue, Shuxinpingxuegong, Qigong, or Daoyinyangshengshu as interventions; (4) at least one of the following metrics was assessed: TC, TG, HDL, LDL, FBG, NO, or ET-1; (5) the experimental group underwent structured exercise training of greater than or equal to 8 weeks duration; and (6) RCTs published in English or Chinese between January 2000 and August 2024 were included. Studies with a clear RCT code or those that mentioned “randomized” and “controlled” in the text were considered as RCTs.

The following types of studies were excluded: (1) repetitively published studies, review articles, conference abstracts, animal experiments, or acute intervention studies that included only a single session of training; (2) articles for which full text was unavailable or for which data were incomplete; (3) studies that did not report in detail on the outcome metrics of interest to the current NMA; (4) studies in which interventions mixed with other therapeutic approaches (eg, novel antihypertensive medications or specific diets); and (5) non-RCTs.

#### Data extraction

Data from each study that met the inclusion criteria were extracted independently by two authors and included basic information about the literature (e.g., lead author, year of publication, country of study), demographic characteristics of the subjects (including sample size, age distribution, and comorbidities in the experimental and control groups), as well as detailed information about the exercise interventions (type of intervention, intensity, duration, frequency, period of the intervention, and whether it was a supervised intervention), and key outcome indicators. If key information was incomplete in some of the studies, the research team offered to contact the authors of the original studies for more detailed data.

#### Risk of bias and GRADE assessment

Utilizing the Tool for the Assessment of Study Quality and Reporting in Exercise (TESTEX) scale, two authors independently evaluated the risk of bias for all eligible studies (Smart et al.). The TESTEX scale is a well-established and reliable instrument specifically designed to facilitate the systematic review of exercise training trials. It comprises 12 items, with the first five items (1 to 5) focusing on the assessment of study quality and the remaining seven items (6 to 12) addressing the assessment of study reporting (Wallin et al.) (Wewege et al.). The scoring range of the TESTEX scale extends from 0 to 15. In the event of discrepancies between the two initial researchers' assessments, they will engage in discussions with other researchers to resolve any differences and reach a consensus. In addition, we systematically assessed the certainty of evidence for outcome indicators using the GRADE framework (Salanti et al.). We applied the GRADE method to the entire network, establishing a framework for systematically rating the certainty of evidence for each paired comparison as high, moderate, low, or very low.

### Quantification and statistical analysis

The present Net Meta-analysis (NMA) combined pre- and post- changes in the experimental and control groups to systematically assess the effects of traditional Chinese mind-body exercise therapy on TC, TG, HDL, LDL, FBG, NO and ET-1.

We also performed a two-by-two Meta-analysis by Review Manager 5.3 software (Nordic Cochrane, Denmark) to assess inter-study heterogeneity (Jackson et al.). To ensure the accuracy of the intervention effects, we used standardized mean differences (SMD) and their 95% confidence intervals (CI) to measure the intervention effect for each indicator, and a random-effects model was used to combine the effect estimates to cope with differences between study participants and intervention protocols (Borenstein et al.). Heterogeneity was quantitatively assessed by the I^2^ statistic and Cochran's Q test, with significant heterogeneity indicated by an I^2^ value greater than 50% or a Q test p ≤ 0.10. To assess publication bias, we used funnel plots for analysis (Higgins et al.). All data analyses were done in STATA 17.0 software (StataCorp LLC, College Station, TX, USA), and the analysis process strictly followed the PRISMA NMA guidelines and implemented multivariate net Meta-analysis of random effects in a frequency-based framework (Salanti) (Bucher et al.). The results of the analysis included the combined effect estimates and their 95% confidence intervals and prediction intervals. The interrelationships among interventions were visualized through network diagrams, where the size of nodes and thickness of connecting lines were proportional to the number of studies, thus visualizing the relative strength and position of each intervention in the network. The network contribution graph further quantifies the contribution of each direct comparison to the overall network, helping to assess the overall impact of each intervention. In order to compare and rank the effectiveness of different campaign interventions, we analyzed them by the cumulative ranked area under the curve (SUCRA) (Salanti et al.). SUCRA values range from 0 to 100, with higher values indicating more effective interventions (Mbuagbaw et al.). The minimal contextualization framework was developed based on the results of SUCRA and GRADE assessments (Brignardello-Petersen et al.).

In addition, publication bias in reticulated Meta-analysis was detected using network funnel plots and visually assessed by the symmetry of the funnel plots.

## Supplemental information

Document S1. Tables S2 and S4 and Data S1

Table S1. Characteristics of the included studies

Table S3. Final classification of six mind-body exercise therapies for hypertensive patients
