# Supplementary material for: Effects of six Chinese mind-body exercise therapies on glycolipid metabolism and vascular endothelial function in hypertensive patients
Source: iScience. 2025 May 15;28(6):112682. doi: 10.1016/j.isci.2025.112682 (PMC12159935; doi:10.1016/j.isci.2025.112682)
Supplement: Document S1. Tables S2 and S4 and Data S1 [file mmc1.pdf]

iScience, Volume *28*

Supplemental information

Effects of six Chinese mind-body exercise therapies on glycolipid metabolism and vascular endothelial function in hypertensive patients

Haojie Li, Xinyu Lin, Xinglu Li, Nan Chen, and Xie Wu

Data S1. PRISMA NMA checklist of items to include when reporting a systematic review involving a network meta-analysis.

Section/Topic	Item #	Checklist Item	Reported on Page #
**TITLE**			
Title	1	Identify the report as a systematic review *incorporating a network meta-analysis (or related form of meta-analysis).*	1
**ABSTRACT**			
Structured summary	2	Provide a structured summary including, as applicable:<br>**Background:** main objectives<br>**Methods:** data sources; study eligibility criteria, participants, and interventions; study appraisal; and *synthesis methods, such as network meta-analysis.*<br>**Results:** number of studies and participants identified; summary estimates with corresponding confidence/credible intervals; *treatment rankings may also be discussed. Authors may choose to summarize pairwise comparisons against a chosen treatment included in their analyses for brevity.*<br>**Discussion/Conclusions:** limitations; conclusions and implications of findings.<br>**Other:** primary source of funding; systematic review registration number with registry name.	1-2
**INTRODUCTION**			
Rationale	3	Describe the rationale for the review in the context of what is already known*, including mention of why a network meta-analysis has been conducted.*	3-4
Objectives	4	Provide an explicit statement of questions being addressed, with reference to participants, interventions, comparisons, outcomes, and study design (PICOS).	4-5
**METHODS**			
Protocol and registration	5	Indicate whether a review protocol exists and if and where it can be accessed (e.g., Web address); and, if available, provide registration information, including registration number.	5
Eligibility criteria	6	Specify study characteristics (e.g., PICOS, length of follow-up) and report characteristics (e.g., years considered, language, publication status) used as criteria for eligibility, giving rationale. *Clearly describe eligible treatments included in the treatment network, and note whether any have been clustered or merged into the same node (with justification).*	5-6
Information sources	7	Describe all information sources (e.g., databases with dates of coverage, contact with study authors to identify additional studies)in the search and date last searched.	6-7
Search	8	Present full electronic search strategy for at least one database, including any limits used, such that it could be repeated.	6-7， Appendix 1
Study selection	9	State the process for selecting studies (i.e., screening, eligibility, included in systematic review, and, if applicable, included in the meta-analysis).	7-8
Data collection process	10	Describe method of data extraction from reports (e.g., piloted forms, independently, in duplicate) and any processes for obtaining and confirming data from investigators.	7-8
Data items	11	List and define all variables for which data were sought (e.g., PICOS, funding sources) and any assumptions and simplifications made.	8-9

**Geometry of the network**	**S1**	Describe methods used to explore the geometry of the treatment network under study and potential biases related to it. This should include how the evidence base has been graphically summarized for presentation, and what characteristics were compiled and used to describe the evidence base to readers.	8-9
Risk of bias within individual studies	12	Describe methods used for assessing risk of bias of individual studies (including specification of whether this was done at the study or outcome level), and how this information is to be used in any data synthesis.	8
Summary measures	13	State the principal summary measures (e.g., risk ratio, difference in means). *Also describe the use of additional summary measures assessed, such as treatment rankings and surface under the cumulative ranking curve (SUCRA) values, as well as modified approaches used to present summary findings from meta-analyses.*	8-9
Planned methods of analysis	14	Describe the methods of handling data and combining results of studies for each network meta-analysis. This should include, but notbe limited to:<br>• *Handling of multi-arm trials;*<br>• *Selection of variance structure;*<br>• *Selection of prior distributions in Bayesian analyses; and*<br>• *Assessment of model fit.*	7-8
**Assessment of Inconsistency**	**S2**	Describe the statistical methods used to evaluate the agreement ofdirect and indirect evidence in the treatment network(s) studied. Describe efforts taken to address its presence when found.	8-9
Risk of bias across studies	15	Specify any assessment of risk of bias that may affect the cumulative evidence (e.g., publication bias, selective reporting within studies).	8-9
Additional analyses	16	Describe methods of additional analyses if done, indicating whichwere pre-specified. This may include, but not be limited to, the following:<br>• Sensitivity or subgroup analyses;<br>• Meta-regression analyses;<br>• *Alternative formulations of the treatment network; and*<br>• *Use of alternative prior distributions for Bayesian analyses(if applicable).*	8-9
**RESULTS†**			
Study selection	17	Give numbers of studies screened, assessed for eligibility, and included in the review, with reasons for exclusions at each stage, ideally with a flow diagram.	9-10
**Presentation of network structure**	**S3**	Provide a network graph of the included studies to enable visualization of the geometry of the treatment network.	11-12
**Summary of network geometry**	**S4**	Provide a brief overview of characteristics of the treatment network. This may include commentary on the abundance of trials and randomized patients for the different interventions and pairwise comparisons in the network, gaps of evidence in the treatment network, and potential biases reflected by the network structure.	,12-13
Study characteristics	18	For each study, present characteristics for which data were extracted (e.g., study size, PICOS, follow-up period) and provide the citations.	10-11，Appendix 2
Risk of bias within studies	19	Present data on risk of bias of each study and, if available, any outcome level assessment.	Appendix 4
Results of individual studies	20	For all outcomes considered (benefits or harms), present, for each study: 1) simple summary data for each intervention group, and 2) effect estimates and confidence intervals. *Modified approaches may be needed to deal with information from larger networks.*	12-14

Synthesis of results	21	Present results of each meta-analysis done, including confidence/credible intervals. *In larger networks, authors may focuson comparisons versus a particular comparator (e.g., placebo or standard care), with full findings presented in an appendix. League tables and forest plots may be considered to summarize pairwise comparisons.* If additional summary measures were explored (such as treatment rankings), these should also be presented.	12-14, Table 2
**Exploration for inconsistency**	**S5**	Describe results from investigations of inconsistency. This may include such information as measures of model fit to compare consistency and inconsistency models, *P* values from statistical tests, or summary of inconsistency estimates from different parts of the treatment network.	11-12, Appendix
Risk of bias across studies	22	Present results of any assessment of risk of bias across studies forthe evidence base being studied.	11-12
Results of additional analyses	23	Give results of additional analyses, if done (e.g., sensitivity or subgroup analyses, meta-regression analyses*, alternative network geometries studied, alternative choice of prior distributions for Bayesian analyses,* and so forth).	Appendix 5 Figure 4-6
**DISCUSSION**			
Summary of evidence	24	Summarize the main findings, including the strength of evidence for each main outcome; consider their relevance to key groups (e.g., healthcare providers, users, and policy-makers).	15-18
Limitations	25	Discuss limitations at study and outcome level (e.g., risk of bias), and at review level (e.g., incomplete retrieval of identified research, reporting bias). *Comment on the validity of the assumptions, such as transitivity and consistency. Comment on any concerns regarding network geometry (e.g., avoidance of certain comparisons).*	18
Conclusions	26	Provide a general interpretation of the results in the context of other evidence, and implications for future research.	18-19
**FUNDING**			Not Applicable
Funding	27	Describe sources of funding for the systematic review and other support (e.g., supply of data); role of funders for the systematic review. This should also include information regarding whether funding has been received from manufacturers of treatments in the network and/or whether some of the authors are content experts withprofessional conflicts of interest that could affect use of treatments in the network.	

PICOS = population, intervention, comparators, outcomes, study design.

* Text in italics indicate wording specific to reporting of network meta-analyses that has been added to guidance from the PRISMA statement.

† Authors may wish to plan for use of appendices to present all relevant information in full detail for items in this section

Table S2. TESTEX scale for analysis study quality of exercise trials

Section	Study quality item scores					Study reporting item scores										
Item	1	2	3	4	5	6a	6b	6c	7	8a	8b	9	10	11	12	
Study	*Eligibility Criteria*	*Randomization*	*Allocation Concealed*	*Baseline data*	*Blinded assessors Primary outcome*	*Outcome Measures Assessed >85% of Participants*	*Adverse events reported*	*Exercise attendance reported*	*Intention-to-treat analysis*	*Between-group Stats Primary outcome*	*Between-group Stats Secondary outcome*	*Outcomes point estimates*	*Control Physical activity*	*Exercise load titrated*	*Exercise volume can be calculated*	Overall TESTEX (/15)
Zhang 2017	1	1	1	1	1	1	1	1	1	0	0	1	0	1	1	12
Shou 2018	1	1	1	1	0	1	1	1	0	1	1	1	0	1	1	12
Xiao 2018	1	1	1	1	1	1	0	0	1	1	1	1	1	0	1	12
Li 2015	1	1	1	1	1	1	1	1	0	1	1	1	0	1	1	14
Li 2019	1	1	1	1	1	1	1	1	1	1	1	1	0	1	1	14
Leung 2019	1	0	1	1	1	1	1	0	1	1	1	1	1	1	1	13
Wen 2021	1	1	0	1	1	1	1	1	1	1	1	1	1	1	1	14
Wu 2023	1	1	1	1	1	1	1	1	0	1	1	1	0	1	1	13
Dong 2020	1	1	0	1	1	1	1	0	0	1	1	1	0	1	1	11
Xie 2014	1	1	1	1	1	1	0	0	0	1	1	1	0	1	1	11
Mao 2006	1	1	1	1	1	1	0	0	1	1	1	1	0	1	1	12
Lin 2017	1	1	0	1	1	1	1	1	0	1	1	1	0	1	1	13
Wang 2021	1	0	1	1	1	1	1	1	1	1	1	1	1	1	1	14
Cheung 2005	1	1	1	1	1	0	1	1	1	0	0	1	0	0	1	10
Zheng 2015	1	1	1	1	1	1	1	1	0	1	1	1	0	0	1	12
Yi 2015	1	0	1	1	1	1	1	0	0	1	1	1	0	1	1	11
Gao	1	1	1	1	0	1	1	1	0	1	1	1	0	1	1	12

2014																
Zheng 2014	1	0	1	1	1	1	1	1	1	1	1	1	0	1	1	13
Thomas 2005	1	1	1	1	0	1	1	1	0	1	1	0	0	1	1	11
Huang 2021	1	1	1	1	1	1	1	0	1	1	1	1	0	1	1	13
Tsai 2003	1	1	1	1	1	1	1	1	1	0	0	1	0	1	1	12
Zhang 2018	1	1	1	1	0	1	1	1	1	0	0	1	0	1	1	11
Chen 2016	1	1	0	1	1	1	1	1	1	1	1	1	0	1	1	13
Xiao 2016	1	1	1	1	1	1	1	1	1	1	1	1	0	1	1	14
Hu 2022	1	1	1	1	0	1	1	1	0	1	1	1	1	1	1	13
Sun 2008	1	1	1	1	1	1	0	0	0	1	1	1	0	1	1	11
Shin 2015	1	1	1	1	1	1	1	0	1	1	1	1	0	1	1	13
Nery 2015	1	1	1	1	0	1	1	1	1	0	0	1	0	0	1	10
Hu 2015	1	1	0	1	1	1	1	1	1	1	1	1	0	1	1	13
Liao 2013	1	1	1	1	1	1	1	1	0	1	1	1	0	1	1	14
Feng 2018	1	1	1	1	0	1	1	1	0	1	1	1	0	1	1	13
Choi 2017	1	1	1	1	0	1	1	1	1	1	1	1	0	1	1	13
Yan 2014	1	1	1	1	1	1	1	0	1	1	1	1	0	1	1	13
Pan 2012	1	1	0	1	1	1	1	1	0	1	1	1	0	1	1	12
Li 2014	1	1	0	1	1	1	1	1	1	1	1	1	0	1	1	13
Chan 2018	1	1	1	1	0	1	1	1	1	1	1	1	0	1	1	13
Studies meeting items criterion(%)	100	89	81	100	69	97	89	72	58	86	86	97	14	89	100	

Table S4. Search strategy of PubMed, Embase, Cochrane, Web of Science, EBSCO, CNKI, WANFANG, VIP.

Database	Search Terms		
	**Mind-body therapies**	**Hypertension**	**Randomized controlled trial**
PubMed [Title/Abstract]	mind-body therapies [MeSH Terms] OR mind-body therapy OR therapies, mind-body OR therapy, mind-body OR mind-body medicine OR Chinese traditional exercise OR Tai Chi OR Tai Ji OR Qigong OR Baduanjin OR Baduanjin exercise OR Wuqinxi OR Wuqinxi exercise OR Yijinjing OR Yijinjing exercise	hypertension [MeSH Terms] OR hypertensive OR arterial hypertension OR blood pressure OR arterial pressure OR diastolic blood pressure OR systolic blood pressure OR high blood pressure* OR blood pressure*, high OR SBP OR DBP	Randomized controlled trial OR randomized OR placebo
Embase [Title/Abstract]	mind-body therapies [Emtree term] OR mind-body therapy OR therapies, mind-body OR therapy, mind-body OR mind-body medicine OR Chinese traditional exercise OR Tai Chi OR Tai Ji OR Qigong OR Baduanjin OR Baduanjin exercise OR Wuqinxi OR Wuqinxi exercise OR Yijinjing OR Yijinjing exercise	hypertension [Emtree term] OR hypertensive OR arterial hypertension OR blood pressure OR arterial pressure OR diastolic blood pressure OR systolic blood pressure OR high blood pressure* OR blood pressure*, high OR SBP OR DBP	Randomized controlled trial OR randomized OR placebo
Cochrane [Title/Abstract/ keywords]	mind-body therapies [MeSH Terms] OR mind-body therapy OR therapies, mind-body OR therapy, mind-body OR mind-body medicine OR Chinese traditional exercise OR Tai Chi OR Tai Ji OR Qigong OR Baduanjin OR Baduanjin exercise OR Wuqinxi OR Wuqinxi exercise OR Yijinjing OR Yijinjing exercise	hypertension [MeSH Terms] OR hypertensive OR arterial hypertension OR blood pressure OR arterial pressure OR diastolic blood pressure OR systolic blood pressure OR high blood pressure* OR blood pressure*, high OR SBP OR DBP	—
Web of Science	mind-body therapies OR mind-body therapy OR therapies, mind-body OR therapy, mind-body OR mind-body medicine OR Chinese traditional exercise OR Tai Chi OR Tai Ji OR Qigong OR Baduanjin OR Baduanjin exercise OR Wuqinxi OR Wuqinxi exercise OR Yijinjing OR Yijinjing exercise	hypertension OR hypertensive OR arterial hypertension OR blood pressure OR arterial pressure OR diastolic blood pressure OR systolic blood pressure OR high blood pressure* OR blood pressure*, high OR SBP OR DBP	Randomized controlled trial OR randomized OR placebo

EBSCO [Abstract]	mind-body therapies OR mind-body therapy OR therapies, mind-body OR therapy, mind-body OR mind-body medicine OR Chinese traditional exercise OR Tai Chi OR Tai Ji OR Qigong OR Baduanjin OR Baduanjin exercise OR Wuqinxi OR Wuqinxi exercise OR Yijinjing OR Yijinjing exercise	hypertension OR hypertensive OR arterial hypertension OR blood pressure OR arterial pressure OR diastolic blood pressure OR systolic blood pressure OR high blood pressure* OR blood pressure*, high OR SBP OR DBP	Randomized controlled trial OR randomized OR placebo
CNKI [Abstract]	Body and mind therapy OR body and mind exercises OR traditional Chinese sports OR traditional Chinese body and mind therapy OR Tai Chi OR Qigong OR Baduanjin OR Wuqinxi OR Yijinjing	Hypertension OR blood pressure OR arterial pressure OR diastolic blood pressure OR systolic blood pressure OR patients with hypertension	Randomized controlled trial OR randomized OR RCT
WANFANG [Abstract]	Body and mind therapy OR body and mind exercises OR traditional Chinese sports OR traditional Chinese body and mind therapy OR Tai Chi OR Qigong OR Baduanjin OR Wuqinxi OR Yijinjing	Hypertension OR blood pressure OR arterial pressure OR diastolic blood pressure OR systolic blood pressure OR patients with hypertension	Randomized controlled trial OR randomized OR RCT
VIP [Abstract]	Body and mind therapy OR body and mind exercises OR traditional Chinese sports OR traditional Chinese body and mind therapy OR Tai Chi OR Qigong OR Baduanjin OR Wuqinxi OR Yijinjing	Hypertension OR blood pressure OR arterial pressure OR diastolic blood pressure OR systolic blood pressure OR patients with hypertension	Randomized controlled trial OR randomized OR RCT
